# Improvement of Oral Bioavailability of Curcumin upon Microencapsulation with Methacrylic Copolymers

**DOI:** 10.3389/fphar.2016.00485

**Published:** 2016-12-21

**Authors:** Donatella Paolino, Ada Vero, Donato Cosco, Tiziana M. G. Pecora, Simona Cianciolo, Massimo Fresta, Rosario Pignatello

**Affiliations:** ^1^Department of Experimental and Clinical Medicine, University of Catanzaro “Magna Græcia”Catanzaro, Italy; ^2^Interregional Research Center for Food Safety and Health, University of Catanzaro “Magna Græcia”Catanzaro, Italy; ^3^Department of Health Sciences, University of Catanzaro “Magna Græcia”Catanzaro, Italy; ^4^Section of Pharmaceutical Technology, Department of Drug Sciences, University of CataniaCatania, Italy

**Keywords:** Eudragit^®^, Retard, microparticles, mucoadhesion, CaCo-2 cells, E12 cells, CLSM

## Abstract

Curcumin (diferuloymethane; CUR) is a yellow pigment used in traditional medicine throughout history for its anti-inflammatory activity. In the last years, the scientific research has demonstrated that CUR effects are related to the modulation of crucial molecular targets, related to several pathologies including cancer, arthritis, diabetes, Crohn’s disease. In this paper, two formulations of microencapsulated CUR obtained by coevaporation with polymethacrylate polymers (Eudragit^®^ Retard) were investigated *in vitro, ex vivo*, and *in vivo*, and results were compared by laser confocal microscopy analysis. The permeation of microencapsulated CUR through CaCo-2 monolayers was evaluated *in vitro*. The mucoadhesion and bioadhesion of the CUR-loaded microparticles were evaluated *in vitro*, using E12 and CaCo-2 human intestinal cells, and *ex vivo*, by means of excised rat intestinal mucosa. After oral administration to rats, microencapsulated CUR showed a sevenfold increase of bioavailability in respect to the neat drug, with a concomitant reduction of the *T*_max_ and a five-fold plasma concentration peak increase.

## Introduction

Curcumin [1,7-bis(4-hydroxy-3-methoxyphenyl)-1,6-heptadiene-3,5-dione; CUR] is the most active component of turmeric (*Curcuma longa*), a perennial herb belonging to the ginger family. It has been used in traditional Indian medicine for about 6000 years to treat wounds, rheumatism and inflammatory diseases (Corson and Crews, 2007; Jurenka, 2009; Leonti and Casu, 2013) but, in the last 50 years, scientific research demonstrated that CUR possesses additional activities such as antioxidant (Soudamini et al., 1992), anti-inflammatory (Abe et al., 1999), anti-atherosclerotic, anti-angiogenesis, and anti-carcinogenic ones (Kunnumakkara et al., 2008). CUR promotes wound healing and muscle regeneration (Panchatcharam et al., 2006), prevents liver injury and kidney toxicity, and exerts medicinal benefits against psoriasis, diabetes, multiple sclerosis, Alzheimer disease, HIV, septic shock, cardiovascular diseases, lung fibrosis, arthritis, and inflammatory bowel disease (IBD), among the most described pathologies (Mecocci et al., 2014; Vecchi Brumatti et al., 2014; Conti et al., 2016). Moreover, CUR demonstrated to successfully inhibit almost every major stage of carcinogenesis, including metastasis (Anand et al., 2008; Liu et al., 2009; Shu et al., 2011).

These wide range of activities are primarily due to the capacity of CUR to modulate various signaling pathways, such as COX-2, MMPs, glutathione, protein kinase C, ATPase, nuclear NF-kb, AP-1, P-gp, MRP-1, MRP-2, ErbB2, α1-acid glycoprotein, Cyclin D1, and others (Anand et al., 2008; Kunnumakkara et al., 2008). Most importantly, many preclinical and clinical studies have confirmed that CUR is significantly non-toxic even at very high doses (Mohanty et al., 2012).

Unfortunately, this compound evidences different problems for its application, such as low-aqueous solubility, rapid systemic clearance, inadequate tissue absorption and degradation at alkaline pH values. As a consequence, its oral bioavailability is extremely poor and CUR can be classified as a class IV compound in the BCS system (Wahlang et al., 2011). Furthermore, the drug is rapidly photodegraded by light, also limiting its clinical use (Cañamares et al., 2006; Kumavat et al., 2016). To overcome these drawbacks, different strategies of micro- and nano-encapsulation of CUR in lipid and polymeric matrices have been investigated (Anand et al., 2007; Bansal et al., 2011; Mohanty et al., 2012; Yallapu et al., 2012, 2014; Lee et al., 2014; Shome et al., 2016; Szymusiak et al., 2016).

Recently, we developed CUR-loaded microparticles made up of Eudragit^®^ Retard copolymers, and we observed that this approach, although was not effective in improving the solubility of CUR in simulated gastric and intestinal fluids, was instead able to protect the drug from UV light-induced chemical degradation (Pecora et al., 2016). Eudragit^®^ Retard resins are poly(ethylacrylate, methyl-methacrylate and chlorotrimethyl-ammonioethyl methacrylate) copolymers, bearing 4.5-6.8% and 8.8-12% quaternary ammonium groups, respectively in the commercial resins called Eudragit RS100 (ERS) and RL100 (ERL). These materials are commonly employed for pH-independent coating of solid oral drug dosage forms; however, since they are insoluble in water at physiological pH values and undergo a certain degree of swelling (Bodmeier and Chen, 1989), they may be suitable for the dispersion and controlled oral delivery of bioactives (Barkai et al., 1990; Pignatello et al., 2001, 2002a,b; Bucolo et al., 2004; Das et al., 2010; Cortesi et al., 2012; Hoobakht et al., 2013).

The bioadhesion is a fundamental prerequisite to be investigated when a drug delivery system is orally administered and it can involve the formation of adhesive bonds between a compound or a material and biological tissues. When the adherent substrate is a mucosal surface, bioadhesion is specifically referred to as mucoadhesion (Shaikh et al., 2011). A common strategy to improve the oral bioavailability of drugs is to use polymers with bioadhesive/mucoadhesive properties, because they may increase the residence time in the gastrointestinal tract.

The aim of this research was to evaluate the *in vitro, ex vivo*, and *in vivo* bioadhesion properties of two novel formulations of CUR-loaded ERL/ERS microparticles, characterized by a different drug-to-polymer ratio (DPR), namely 1:5 or 1:10. The bioadhesion of CUR microparticles was investigated by confocal laser scanning microscopy (CLSM) with respect to the free drug on CaCo-2 cells and the permeation profile of the drug was evaluated by using a monolayer of the same cell line. Moreover, HT29MTXE12 (E12) cell line, a goblet cell-like subclone of the human colon carcinoma HT29 cell line, which is able to secrete an adherent mucus layer (Navabi et al., 2013), was used to evaluate the microparticle mucoadhesion. The latter property was also investigated by an *ex vivo* model of rat gut sac. Finally, the *in vivo* bioavailability of CUR following the oral administration of the drug as free form or in the microparticles was assessed.

## Materials and Methods

### Materials

CUR (Curcuma longa rhizome dry extract; total curcuminoids 95% min.) was produced by Vivatis Pharma GmbH (Hamburg, Germany) and was kindly gifted by Labomar srl (Istrana, Italy); ERL and ERS resins (Evonik Rohm GmbH, Darmstadt, Germany) were kindly provided by Rofarma Italia srl (Gaggiano, Italy). CaCo-2 epithelial cells were obtained from the Zooprophylactic Institute of Lombardia and Emilia Romagna; E12 cells were purchased from Sigma-Aldrich Chimica srl (Milan, Italy). HPLC solvents were purchased from VWR PBI International (Milan, Italy); all other materials and solvents used throughout this investigation were of analytical grade (Carlo Erba, Milan, Italy).

### Production of CUR Microparticles

The Eudragit^®^ resin blend (ERL/ERS, 30:70, w/w) (2 mg total) was dissolved in 20 ml of ethanol by overnight mechanical stirring at room temperature. The amount of drug required to obtain the chosen DPR weight ratios (1:5 or 1:10, for batches **CEM1** and **CEM2**, respectively) was dissolved in 10 ml acetone and added to the polymer solution. The mixture was then slowly dropped, within 60 min, through a glass burette into 50 ml distilled water containing 0.02% (w/v) Tween^®^ 80, under mechanical stirring at 150 rpm and room temperature, to produce the microparticles. The suspension was left stirring at room temperature for about 24 h to allow the evaporation of the solvents; the final polymer concentration was 4% (w/v). The product was then frozen at -20°C for 12-16 h and lyophilized for 24 h (Edward Modulyo).

The solid samples were finally passed through European Pharmacopoeia standard metallic sieves using a vibratory apparatus (Giuliani Tecnologie srl, Turin, Italy), to collect the microparticle sieve fractions between 420 and 125 μm (40 to 120 mesh).

Details of physico-chemical characterization of the obtained microparticles have been previously described (Pecora et al., 2016).

### Cell Cultures

E12 cells and Caco-2 cells were cultured in Dulbecco’s Modified Eagles Medium (DMEM) at 37°C (5% CO_2_). Cells were seeded using D-MEM medium with glutamine, penicillin (100 UI/mL), streptomycin (100 μg/mL), amphotericin B (250 μg/mL), and FBS (10% v/v).

### Permeation Experiments through CaCo-2 Cells

*In vitro* permeation studies were performed using CaCo-2 epithelial cells used between passage 21 and 38. They were seeded at a density of 10^4^/cm^2^ into Snapwells^TM^ (1 cm^2^, 0.4 μm pore size; Costar Corporation, Milan, Italy). The growth media was replaced every day. Confluent cell monolayers were obtained 18–22 days after seeding. The confluence was demonstrated by means of the *trans*-epithelial electrical resistance (TEER) measured with a voltmeter (Millicell ERS). The measured TEER was between 500 and 600 cm. Permeation experiments were performed by means of Franz type diffusion cells, putting the Snapwells^TM^ containing the CaCo-2 cells monolayer on the donor compartment. An aliquot of 200 μl of a suspension of neat CUR in PBS (pH 7.4) (1 mg/ml, corresponding to 2.7 mM) or of each formulation (**CEM1** or **CEM2**) containing an equivalent amount of drug was applied to the monolayer. The acceptor phase was PBS (pH 7.4) containing 0.5% (w/v) hydroxypropyl-β-cyclodextrin (HP-β-Cyd) as a solubilizing agent to facilitate CUR permeation. Samples from the receiving solution were withdrawn at different times during the experimental period (6 h); the sample volumes were immediately replaced with the same amounts of fresh HP-β-Cyd solution. At the end of the experiments the TEER was re-measured to check the cell monolayer integrity. All samples were analyzed by HPLC to determine the concentration of CUR. The obtained values were corrected for the dilution used during sampling.

### Confocal Laser Scanning Microscopy (CLSM)

The interaction between the CaCo-2 cells and CUR formulations was also evaluated by CLSM studies. Briefly, the cells were placed in 6-well culture plates (4 × 10^5^ cells/ml) containing a sterile glass slide. Successively, the cells were treated with CUR (100 μM) as the free drug or loaded microparticles and incubated for 6 and 24 h. After incubation, each well was washed three times with PBS to remove the excess of drug/formulations and cells were fixed on the sterile glass as previously described (Cosco et al., 2015). The CLSM analysis was carried out using a laser scanning confocal microscopy (CLSM) Leika TCS SP2 MP at λ_exc_ = 440-470 nm and λ_em_ = 480-519 nm for CUR and at λ_exc_ = 405 nm, and λ_em_ = 460 nm for Hoechst probe used with the aim of evidencing the nuclei.

A scan resolution up to 1024 × 1024 pixels with an Ar/Kr laser beam of 75 mW, equipped with a 488/405 analyzer filter, was used for experimental investigations. Samples were recorded by a macro developer software package having multi-dimensional series acquisition and direct access digital control knobs. An immersion oil lens 63× was used, with a 2× magnification.

### HPLC Determination

The HPLC analysis was performed according to literature (Tønnesen et al., 1986; Pecora et al., 2016) on a Hewlett-Packard 1100 chromatograph equipped with on-line diode array detector (DAD) and a Kontron SFM fluorescence detector (FLD). The freeze-dried samples were redissolved in 1 ml HPLC-grade methanol and injected into a 20-μl loop combined with an analytical C18 Chrompack Inertsil ODS-2 column (Varian, 4.6 mm × 250 mm, 5 μm) shielded by a pre-column (4.6 mm × 4.5 mm). Chromatograms were generally recorded using FLD (λ_exc_ 422 nm, λ_em_ 557 nm) and DAD (absorbance centered at 420 nm). An isocratic flow rate of 1 ml/min of methanol/water (96:4, v/v) was employed. A freshly prepared CUR standard in methanol (0.1 mg/ml) was used to create a calibration curve (linear in the range 1-8 μg/ml; *r*^2^ = 0.988).

### *In Vitro* Evaluation of Bioadhesion and Mucoadhesion

CaCo-2 and E12 monolayers were rinsed with HEPES-buffered DMEM and equilibrated in the same medium at 37°C for 60 min. The cells were seeded at a density of 2 × 10^4^ cells/filter on onto Transwells^TM^ polycarbonate membrane inserts (1 cm^2^, 0.4 μm pore size: Costar Corporation, Milan, Italy). Confluence was reached in 24 days. At the end of this period, 0.5 ml of **CEM1** and **CEM2**, or a suspension of CUR (1 mg/ml) in PBS (pH 7.4) were added to the apical side of the monolayer at two drug concentrations (0.1 or 0.01 mg/ml). Monolayers were incubated at 37°C in a Titramax 1000 shaking incubator for 60 min at 100 rpm. The formulations were then aspired and monolayers were washed three times with the incubation buffer. CUR was quantified by HPLC and the results were expressed as percentage of the applied dose.

### *Ex Vivo* Mucoadhesion Assay

Male Wistar rats (290–340 g) were purchased from Harlan, Italy (Correzzana, Milan, Italy). Animal experiments followed the main procedure outlined by the local Ethics Committee, Italian law and the accepted international standards for biomedical research. Care and handling of the animals followed the EEC Council Directive 86/209, recognized and adopted by Italian Government (D.M. 95/2003-D). Rats were fasted overnight before euthanasia by cervical dislocation. The intestine was removed after the incision, and the jejunum was cut in 5-cm pieces, everted, and one of its ends sutured; the resulting sac was filled with saline. The sacs were introduced into tubes containing the formulation under analysis (0.1 mg/ml) and incubated under stirring for 60 min. The sacs were then removed and washed sequentially four times with a total of 5 ml of medium; the washes were pooled and assayed by HPLC. Results were expressed as the percentage of the applied CUR dose.

### Oral Bioavailability

Male Wistar rats (290–340 g) were housed in a temperature- (23 ± 1°C) and light-controlled room (12 h light/dark cycle). They were allowed ad libitum access to food and water and commercial for 7 days. Rats were randomly divided into three groups of eight animals each. The control group was given neat CUR as a suspension in PBS (pH 7.4) (at 1 mg/ml drug concentration). The second and third groups were treated with **CEM1** or **CEM2**, respectively. The CUR dose in all groups was fixed at 100 mg CUR/kg b.w. All the formulations were orally administered by oral gavage by mean of flexible plastic tubes of the length of 5 cm. At pre-determined interval (0, 30, 60, 90, 120, 180, 240, 300, and 360 min) after administration, blood was harvested from the abdominal Vena Cava of rats under light anesthesia (diethyl ether) and placed into heparinized tubes to prevent clotting. Plasma was immediately prepared by centrifugation at 1000 × *g* for 15 min at 4°C and stored at -80°C until analysis. Plasma (200 μL) was mixed with 200 μL of 0.1 M sodium phosphate buffer (pH 6.8) containing 0.1% EDTA, 200 μL of distilled water, and 600 μL of methanol. The mixture was vortexed for 3 min and, after the addition of 4 mL of hexane, was shaken vigorously and centrifuged at 1000 × *g* for 10 min at 4°C. The hexane layer was discarded and 1.2 mL of distilled water and 3 mL of ethyl acetate were added to the mixture (aqueous-methanol layer). This was shaken vigorously and centrifuged at 1000 x g for 15 min at 4°C, and the ethyl acetate layer was finally collected. This ethyl acetate extraction was repeated four times. The combined extraction phases were evaporated to dryness *in vacuo* and the residue was dissolved in 100 μL of methanol to be analyzed for CUR content by HPLC, as described above.

Pharmacokinetic calculations were carried out using WinNonlin Professional, version 5.3 (Tharsight, Mountain View, CA, USA).

### Statistical Analysis

Experimental data were analyzed by one-way analysis of variance. *A posteriori* Bonferroni *t*-test was done to check the analysis of variance test. A *P*-value < 0.05 was considered to be statistically significant. Values are reported as the mean ± SD.

## Results And Discussion

Curcumin (diferuloymethane) is a natural compound, possessing a potent antioxidant activity, and other biologically interesting effects (Perrone et al., 2015), but whose therapeutic use is strongly limited by the very low solubility and oral bioavailability (it belongs to class IV of the Biopharmaceutical Classification System, BCS), along with a rapid metabolism into inactive metabolites and rapid clearance from the body (Anand et al., 2007). Furthermore, at neutral or basic (i.e., intestinal) pH values, CUR becomes rapidly inactivated (Wang et al., 1997).

Daily doses up to several grams are then usually recommended for pure CUR or turmeric in order to reach active serum concentrations of the active compound. Due to the large therapeutic and commercial (nutraceutical) interest toward CUR, various strategies have been exploited and sometime marketed to make CUR more soluble and absorbable through the gut: along with the complete range of lipid- and polymer-based micro- and nanocarriers (micelles, liposomes, nanoparticles, SLN, etc.) (Dutta and Ikiki, 2013; Kurita and Makino, 2013; Prasad et al., 2014; Shome et al., 2016; Szymusiak et al., 2016), also chemically-driven solutions have been attempted, such as phytosomes (Kidd, 2009), molecular dispersions in hydrophilic polymers or gels, blending with adjuvants, up to chemical modifications to the molecule of the drug (Sehgal et al., 2011). Evidence of *in vivo* enhanced bioavailability in human subjects for a CUR nanoparticle formulation (Theracurmin^®^) has been recently reported (Kanai et al., 2012). Another recently patented technology (Enterosoma^TM^, Labomar srl) has been exploited to enhance gut absorption and systemic bioavailability of CUR. This technology is based on a gastro-resistant tablet whose core contains a combination of chitosan salified with N-acetylcysteine (NAC) and a polysorbate, that work in synergy to reduce the intestinal and hepatic degradation of naturally-derived active ingredients (Labomar, 2013).

In this research, we evaluated in different biological models two formulations of CUR microencapsulated with a 30:70 ERL/ERS blend, and characterized by a 1:5 (batch **CEM1**) or 1:10 DPR value (batch **CEM2**). They correspond to samples D42 and D43 in the paper describing their preparation (Pecora et al., 2016).

The intestinal drug absorption of a drug depends on various factors, such as the intrinsic stability of the compound in the intestinal lumen, the metabolic stability towards the enzymes present within the gut wall/mucosa, the presence of transporters, when they are involved in the absorption or expulsion of the considered drug molecules.

Drug permeability across the epithelial mucosa and the gastro-intestinal transit time of the dosage form can be considered among the most relevant of these factors. In the case of CUR, its low permeability has been justified by considering an extensive intestinal first-pass metabolism and accumulation inside gut wall cells (Wahlang et al., 2011). Thus, based on its poor aqueous solubility and intestinal permeability, CUR is usually classified as a BCS Class IV molecule.

Therefore, to assess the performance of the proposed microparticle formulations, the permeation of loaded CUR through a CaCo-2 cell monolayer was tested *in vitro*, along with the microparticle mucoadhesion features; the latter property was also evaluated *ex vivo* in an inverted rat gut assay. *In vivo* bioavailability of CUR microparticles was finally evaluated in rats.

### Permeation Experiments Through CaCo-2 Monolayers

An important parameter to be considered for a drug administered orally is the absorption through the intestinal mucosa. An interesting, preliminary test that may be predictive is the evaluation of the permeation through a monolayer of CaCo-2 cells. These cells in fact possess morphological characteristics proper of small intestine cells, such as tight intercellular junctions and microvilli. Moreover, they express intestinal enzymes (aminopeptidases, esterases, sulfatases, and cytochrome P450 enzymes) and transporters (e.g., bile acid carrier, large neutral amino acid carrier, biotin carrier, monocarboxylic acid carrier, PEPT1, and *p*-glycoprotein) (Ng and Borchardt, 1993).

**FIGURE 1:**
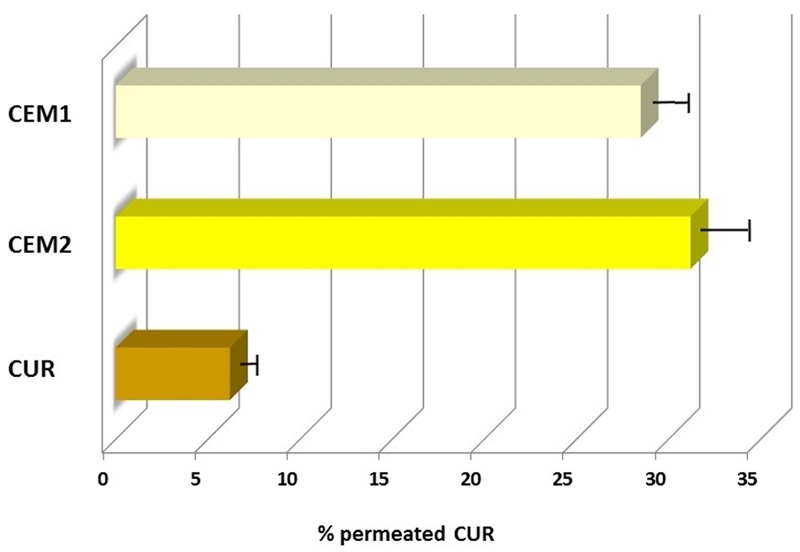
**Permeation profiles of neat or microencapsulated CUR (**CEM-1** and **CEM-2** formulations) through CaCo-2 cells monolayers**.

The microparticles containing CUR and free CUR were thus tested on CaCo-2 monolayers. As reported in **Figure 1**, both the examined microparticle batches showed an enhancement of CUR permeation compared to the neat drug. At the end of the experiment, **CEM1** and **CEM2** microparticles showed a CUR permeation of 31.2 and 28.5%, respectively, compared to 6.2% for the neat drug, corresponding to 5- and 4.6-fold increase, respectively. No significant variation was observed between the two microparticles formulations, which differ for the weight ratio between CUR and the copolymers (DPR value).

### *In Vitro* Evaluation of Bioadhesion and Mucoadhesion

To improve the permeability of a drug following oral administration, a common strategy is to prolong its residence time using a mucoadhesive carrier, that would increase the gastro-intestinal tract residence time and finally lead to an improvement of drug oral bioavailability.

To assess the bioadhesive properties of ERL/ERS microparticles, *in vitro* cell culture models of human intestinal epithelium were used. CaCo-2 cells, when cultured onto a suitable membrane, spontaneously differentiate into monolayers of polarized enterocytes, connected by tight junction; these cells lack mucus-secreting goblet cells, therefore they represent a useful model to evaluate the bioadhesive properties of a drug formulation.

**FIGURE 2:**
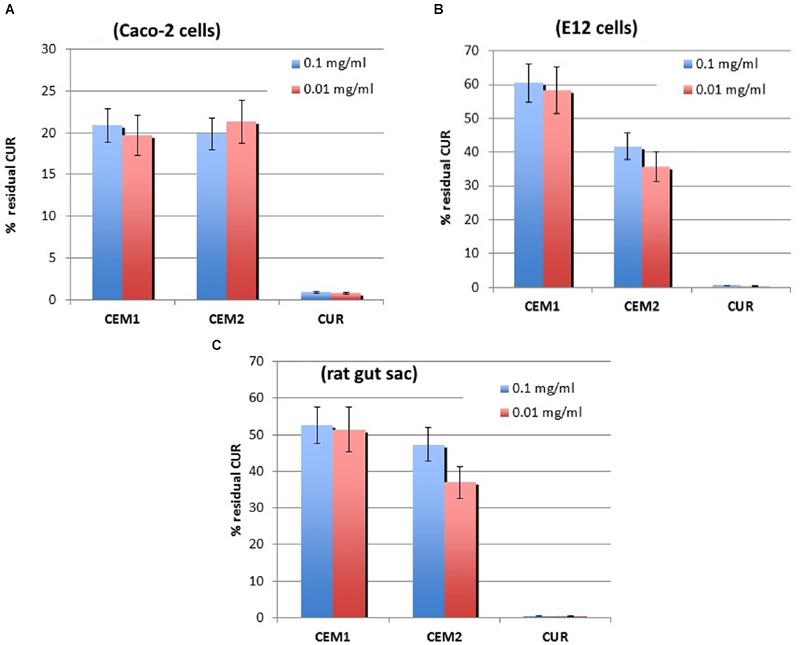
**Experimental results from bioadhesion studies.** *In vitro* (**A** and **B**) and *ex vivo* **(C)** mucoadhesion assay of neat CUR or microencapsulated in **CEM-1** and **CEM-2** formulations. All samples were assayed at two drug concentrations (0.1 or 0.01 mg/ml). Values are the mean (SE) of three to four experiments.

In our experiments, both **CEM1** and **CEM2** demonstrated a good bioadhesion with the cellular support. In fact, a significant increase of the amount of CUR present on the surface of CaCo-2 monolayer was registered (about 20%), whereas the neat CUR gave a residual amount of less than 1% of the applied dose. Also in this case, no significant difference was observed between two different microparticle formulations and the two different tested dilutions of each microparticle suspension (**Figure 2A**).

**FIGURE 3:**
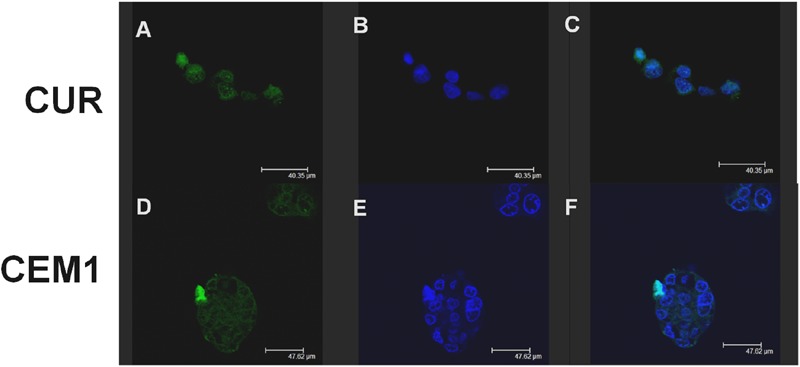
**Confocal laser scanning microscopy micrographs of CaCo-2 cells treated with free CUR or **CEM1** microparticles after 6 h incubation: CUR filter (A,D)**, Hoechst filter **(B,E)**, merge **(C,F)**.

**FIGURE 4:**
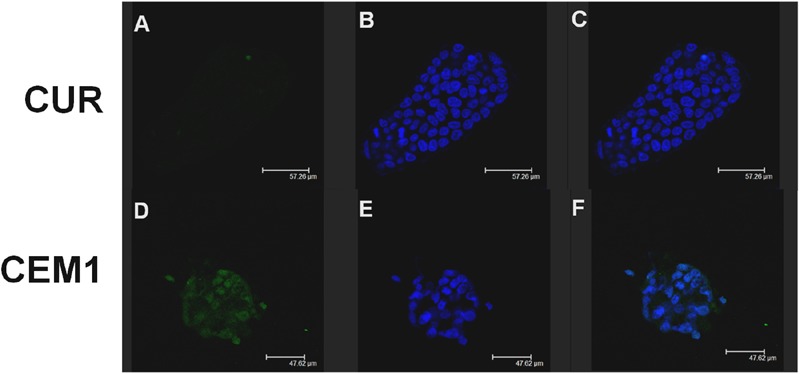
**Confocal laser scanning microscopy micrographs of CaCo-2 cells treated with free CUR or **CEM1** microparticles after 24 h incubation: CUR filter (A,D)**, Hoechst filter **(B,E)**, merge **(C,F)**.

Confocal laser scanning microscopy experiments confirmed this trend: namely, **CEM1** evidenced a stronger interaction with CaCo-2 cells with respect to the free form of the active compound after 6 h incubation. In fact, in **Figure 3**, it is possible to observe a more evident fluorescence of cells when they were incubated with the microparticles containing the drug, thus confirming the bioadhesive properties of these systems. This thesis was confirmed by the investigation of the interaction rate between CUR microparticles and CaCo-2 after 24 h incubation; in particular, the fluorescence of cells incubated with neat CUR was dramatically decreased, probably as a consequence of metabolism phenomena and cell surface desorption of the drug, while microparticles allowed to maintain a certain cell fluorescence (**Figure 4**). Similar results were obtained with **CEM2** (data not shown), and the described cell interaction profiles are in very good agreement with the experimental work performed by Wahlang et al. (2011), who demonstrated that CUR is poorly permeable across CaCo-2 cells, due to its chemical and biological degradation.

Even more clear was the difference in the *in vitro* mucoadhesive capacity of CUR microparticles compared to the free drug, using a cell line secreting mucin (E12 cells). Monolayers of E12 cells can develop a mucus gel layer of approximately 150 μm (Navabi et al., 2013). This layer measure corresponds to that one of living human upper bowel. The above mucus works like a significant barrier against permeation of lipophilic compounds; furthermore, this cell line expresses mucins MUC1 e MUC2, that are located in upper bowel and are implicated in host-pathogen relationships. Thanks to these properties, the E12 cell monolayer cultures represent a suitable *in vitro* model to evaluate the degree of polymer and drug carriers mucoadhesion (Keely et al., 2005).

Using this model, the two tested microparticle formulations allowed a drug retention of approximately 60 and 40%, respectively for the microparticles produced using a DPR value of 1:5 and 1:10, whereas neat CUR did not show any mucoadhesive capability in the assay conditions (**Figure 2B**). Also in this case, no relevant difference between the two tested drug concentrations was registered.

### *Ex Vivo* Evaluation of Mucoadhesion

To confirm the results obtained with cell models, further experiments were realized on the same specimens using the inverted bowel model. Interestingly, the *ex vivo* assay produced overlapping results with the *in vitro* test on E12 cells, in terms of CUR percentage remained absorbed in the mucus layer (**Figure 2C**). These results demonstrated the reliability of these experimental models (Keely et al., 2005) and confirmed the mucoadhesive features of Eudragit^®^ microparticles, although these polymers are usually not considered bioadhesive materials, at least at the level of chitosan or alginates. Our experimental data showed that these micronized resins ensure an efficient entrapment inside the bowel epithelial cells mucus layer and also an interaction with mucin non-secreting cells (CaCo-2).

These findings support their potential role as biomaterials to obtain DDS suitable for an efficient controlled oral drug release (Pignatello et al., 1997, 2002a,b).

### Oral Bioavailability

**FIGURE 5:**
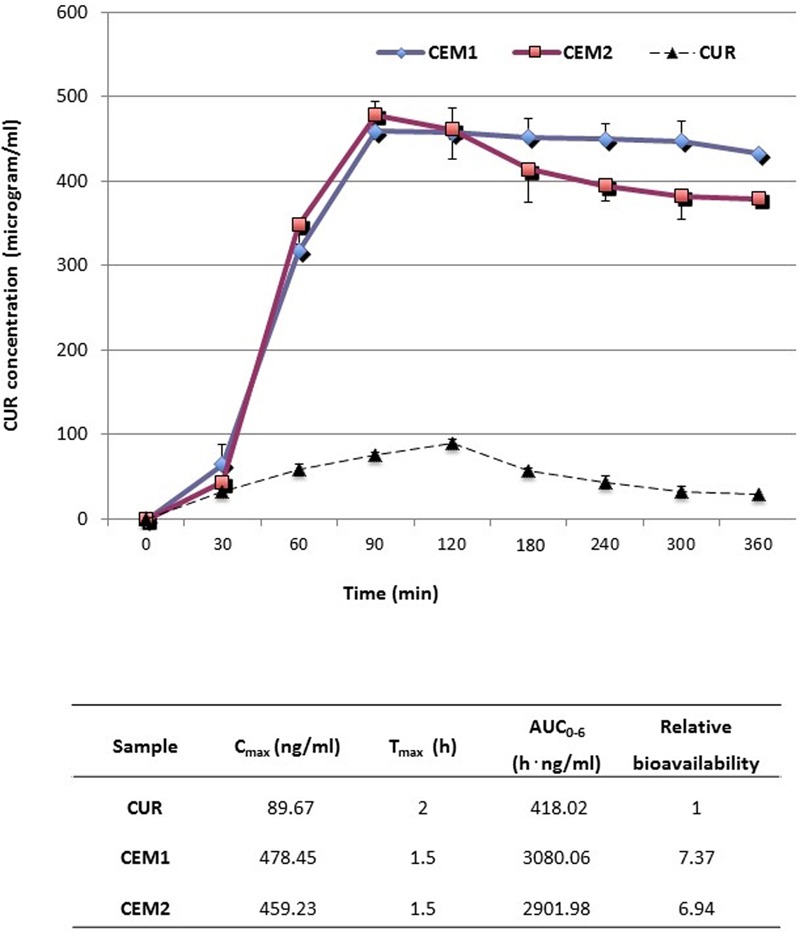
**Plasma concentration/time curves and mean pharmacokinetic parameters obtained after oral administration (gastric intubation) to rats of neat or microencapsulated CUR.** Each test group was composed by eight animals.

The mean CUR plasma concentration–time profiles after oral gavages of 100 mg/kg b.w. of CUR as an aqueous suspension or CUR-loaded microparticles **CEM1** and **CEM2** are expressed in **Figure 5**, along with the relevant pharmacokinetic parameters.

After oral administration, neat CUR is characterized by a relatively fast absorption, after which the drug plasma concentration decreased rapidly, due to distribution and metabolization, resulting in a short t_1/2_ (approximately 1.32 h) (Reza Kheiri Manjili et al., 2016). In our experiment, CUR was still detectable in plasma after 6 h, in accordance with literature data (Reza Kheiri Manjili et al., 2016), although at very low concentrations.

Conversely, both the tested microparticle formulations increased by about sevenfold the oral bioavailability of CUR [in terms of plasma AUC(0-6 h)]. A simultaneous fivefold increase of the maximum experimental plasma concentration (*C*_max_) and reduction of the corresponding peak time (*T*_max_) from 120 to 90 min were registered (**Figure 5**). In particular, the increase of *C*_max_ obtained after the oral administration of microparticles was not far from the improvement recently registered upon multiple oral administration of CUR-loaded polymeric nano-sized micelles (Reza Kheiri Manjili et al., 2016). Most noteworthy, CUR plasma levels after the administration of microparticles remained at values just below the *T*_max_ (400-450 μg/ml) for the entire duration of the experiment (6 h) (**Figure 5**), indicating a sustained release of the drug from the polymeric carrier and a prolonged gastro-intestinal absorption.

From a technological point of view, no significant difference between the two tested microparticle batches were observed. This suggests that the encapsulation of CUR within these polymeric matrices allowed similar features *in vivo*, in terms of drug release and adsorption, regardless from the drug and copolymers weight ratio and composition.

To correctly appraise these experimental data, it must be considered that microencapsulation of CUR in the ERL/ERS matrix had shown to induce to a micronization or partial amorphization of CUR, as suggested by solid-state analysis (Pecora et al., 2016). Such a physico-chemical change could have led to an increase of gastro-intestinal adsorption of the drug. More specifically, by considering the registered *T*_max_ values, it can be supposed that a large part of the drug was released and absorbed at the level of stomach.

From the *in vivo* experimental results, the higher *C*_max_ and larger AUC obtained indicated that the microparticles can sustain the release and absorption of CUR, prolong the drug transit time, prevent a rapid metabolism and inactivation of the entire dose of given drug, and thus enhance its bioavailability.

## Conclusion

Enhancement of the oral absorption profile and bioavailability of CUR is highly required to convey this promising active compound in the near future to the vanguard of therapeutic agents for clinical treatment of human diseases.

Different technological and chemical strategies have been until recent proposed to make CUR more water soluble and absorbable in the gastro-intestinal tract. Among the possible tools, polymer-based microencapsulation shows some useful features, in terms of costs, stability, easy industrial production and scaling-up, as well as regulatory acceptance.

In this study we have tested with different *in vitro, ex vivo*, and *in vivo* models two Eudragit^®^ Retard microparticle formulations containing CUR, which had previously shown interesting technological characteristics and the ability of protecting this drug from photodegradation (Pecora et al., 2016). Microparticles showed good muco- and bioadhesive properties, which reflected into an improved *in vivo* oral bioavailability of CUR, ensuring higher plasma concentrations of the drug for a longer time, than those measured after the administration of the neat drug, and thus advocating a prolonged clinical significance of this drug.

Confocal laser microscopy studies confirmed that microencapsulation was able to facilitate the interaction of CUR - which exists in an amorphous or micro-crystalline form within the polymer matrix - with gut wall cells.

The positive data collected in this study will prompt to further investigate CUR-loaded Eudragit^®^ microparticles in different models of human pathologies, such as inflammatory bowel disease and colon cancer.

## Author Contributions

RP and TP ideated and carried out the whole project on microparticles loaded with natural antioxidants. SC participated to the synthesis of CUR-loaded microparticles. DP, AV, and MF planned and performed the biological studies. DC collaborated with the biological experiments and carried out the CLSM studies.

## Conflict of Interest Statement

The authors declare that the research was conducted in the absence of any commercial or financial relationships that could be construed as a potential conflict of interest.
